# Medication-Related Experience of Deaf American Sign Language Users

**DOI:** 10.3928/24748307-20231116-01

**Published:** 2023-10

**Authors:** Mariam Paracha, Ellen Wagner, Olivia Brumfield, Jonah Winninghoff, Jordan Wright, Jason Rotoli, Peter Hauser

## Abstract

**Background:**

Previous studies showed that deaf and hard-of-hearing (DHH) individuals have low health literacy related to prescription labels. This study examined the DHH's experience with understanding prescription labels and how technology can impact that experience.

**Objectives:**

The purpose of this qualitative study was twofold: (1) gain a more enhanced understanding of DHH experiences in understanding prescription labels with a focus on language needs, expectations, and preferences, and (2) assess the potential role of technology in addressing the communication-related accessibility issues which emerge from the data.

**Methods:**

In this study, 25 Deaf American Sign Language users who picked up a prescription from a pharmacy within the past year were interviewed. A thematic analysis, which included a systematic coding process, was used to uncover themes about their experiences picking up and using prescription medications.

**Key Results:**

Thematic analyses identified that medication-related experiences centered around themes: (1) medication information seeking; (2) comfort taking medication; (3) picking up medication; and (4) communication with the pharmacy team. A large contributor to the communication experience was the perception that the pharmacist was not being respectful. Regarding comfort taking medications, 12% of participants expressed a lack of understanding medications while taking medication. This led to participants largely using online resources when seeking medication information. This study also found that technology greatly aided the participants during this experience.

**Conclusion:**

This study recorded the experiences within the context of limited health literacy and aversive audism found that the DHH individual repeatedly encountered communication barriers, which may contribute to their poor medication literacy. Thus, future studies should explore how to leverage the potential benefits of technology to improve the pharmacy experience of the DHH, thereby improving medication literacy. [***HLRP: Health Literacy Research and Practice*. 2023;7(4):e215–e224.**]

## Plain Language Summary

Previous studies have shown that deaf and hard-of-hearing (DHH) individuals have low health literacy and higher rates of unintentional medication misuse. DHH participants described their experiences related to the pharmacy and technology, as situated around negative attitudes and language barriers. Based on four themes, which emerged from our analysis, we identified areas where may help to reduce these care inequities.

The deaf and hard-of-hearing (DHH) community has a wide range of language preferences and hearing statuses. Unlike individuals with hearing loss who identify as deaf (represented by lower case [d]), the American Sign Language (ASL) user who is Deaf (represented by upper case [D]) is part of a social-linguistic community with a unique culture and common language, ASL. As seen in other marginalized groups, DHH individuals face significant challenges due to existing disparities in health literacy and accessing medical services and are sevenfold more likely to have inadequate health literacy (McKee et al., 2015). Deaf individuals are twice as likely to visit emergency rooms as result of delayed care and their visit is further impacted by barriers of communication, language deprivation, and low literacy skills (McKee et al., 2015; McKee et al., 2019). As a result, DHH adults are a medically underserved group with suboptimal health outcomes compared to the general population. Previous studies have shown that individuals with limited proficiency or reduced-communication abilities are at a high risk for experiencing health disparities (McKee et al., 2015; McKee et al., 2019). Such disparities are rooted in a systematic lack of general literacy which further compounds the issue when extended to health literacy, as DHH adults generally read and write at the elementary-to-middle school level (Allen, 1986; Berkman et al., 2010; Pollard & Barnett, 2009; Traxler, 2000; Zazove et al., 1993).

Up to 48% of DHH adults have basic or below-basic health literacy compared to 36% of adults in the general population (McKee et al., 2015). This may have originated from a lack of incidental learning opportunities on health-related issues, having never overheard discussions of their family's medical history, or listening to the radio and television commercials on health issues; this is further compounded by limited internet literacy and the ability to deduce information based on reduced funds of world knowledge (Chong et al., 2019). This gap in health-related knowledge and understanding often leads DHH adults to search for alternative sources of health information such as health websites, books, trusted peers, or family members for better understanding of their general health (Kushalnagar et al., 2018; McKee et al., 2015).

Access to medication information is a primary concern for the DHH community, especially relating to understanding prescription labels. For example, despite information provided with medications such as acetaminophen and aspirin, many participants in the McKee et al. (2011) study remained confused about which was used for cardioprotective purposes. The ability to access medication information is also impaired due to language discordance and subsequent communication barriers with providers and pharmacists.

Prescription labels must be written in plain language. Ferguson and Liu (2015) suggested that the rate of unintentional medication misuse was 33%; however, the actual rate of unintentional medication misuse due to misunderstanding could be higher especially considering this study population included 40% of participants who completed a college degree, which is higher than the general Deaf population in the United States. Participants who were taking several medications expressed concerns about safety and related their experiences with nonadherence, medication errors, and uncertainty about generic drugs (Chong et al., 2021). DHH participants had to learn from hands-on experience using their given medications correctly after suffering adverse side effects. Sadly, no participants considered contacting their pharmacists after these adverse events (Ferguson & Liu, 2015).

Most pharmacists are not prepared to communicate effectively with DHH adults; however, improving communication between pharmacists and patients has the potential to increase medication adherence and decrease unintentional misuse (Ngoh, 2009). Most DHH adults are not proficient in English, as ASL is their common language (McKee et al., 2015; McKee et al., 2019). The language barrier not only frustrates DHH adults but also has the potential to create issues with unintentional medication misuse due to miscommunication, ultimately leading to subpar health outcomes when compared to hearing cohorts. Pharmacists believed that in many of their encounters with DHH individuals, it was a matter of guessing what patients were trying to convey, and pharmacists attributed this situation to their lack of experience with DHH individuals. Although writing was the preferred communication method with their DHH individuals, it is dependent on patient being literate, which is unreliable. Additionally, the presence of an interpreter was useful, but there were concerns with the accuracy of translation (Chong et al., 2019).

Current DHH health literacy research has shown the potential of technology to facilitate access to health-related information. The most important dissemination tools for DHH participants were ASL-accessible health videos and medical websites (McKee et al., 2011). The websites (www.deafmd. org and www.deafdoc.org) offered videos containing information about general health or medical terminology in ASL format, which is most accessible for participants with native ASL. Public health studies reported that social media is also a promising avenue to educate general users on health information and health care services. Therefore, social network sites that incorporate ASL videos afford the opportunity to share health information and stimulate online interaction between DHH adults (Kushalnagar et al., 2018). Moreover, pharmacists reported that a mobile application could potentially save time and allow for a more efficient dispensing process for DHH adults (Ryan & Kushalnagar, 2018). This type of platform could indirectly educate health care professionals on Deaf culture and improve their attitude toward the DHH community. Three mobile health apps used by DHH individuals are SignSupport, iSignIT, and Ava. These provide access to interpreters and live captioning (App-Synopsis, 2013; Ava, 2020; Chininthom, 2020). However, none of these apps harness data to improve health outcomes and make informed decisions about the medications.

The thematic analysis to explore the medication-related experiences of the DHH adults who are fluent in ASL is novel. At present, the understanding of their technology and medicines uses, medication-related behaviors, and relationships with pharmacy teams is limited. The purpose of this pilot qualitative study, therefore, is twofold: (1) gain a better understanding of DHH experiences in dealing with prescription labels with a focus on language needs, expectations, and preferences, and (2) assess the potential role of technology in addressing the communication-related accessibility issues from the data.

## Methods

### Recruitment

Participants were recruited primarily via posts in Deaf-specific Facebook groups, through flyers posted on bulletin boards across a university campus in the Northeast, and via the distribution of flyers among various organizations within the New England area Deaf Community using a purposive sampling approach. In addition, social media posts were made every other week. Participants contacted the first author via an email address provided on the flyers and in social media posts. Those interested in participating completed a brief screening process to satisfy the inclusion criteria: 18 years or older, identify as Deaf, use ASL as their primary language, and must have picked up a prescription from a pharmacy within the past year. Individuals were excluded if they used spoken language, unable to consent for themselves, or worked as a Deaf healthcare professional. Once eligibility was established, an information sheet was emailed to the participant to schedule an interview and confirm communication preferences. We conducted interviews until: (1) data saturation and redundancy were reached (i.e., no additional significant themes emerged from the interviews) and (2) meaning saturation was reached (i.e., a context-rich and nuanced understanding was developed) (Thorogood & Green, 2013; Morse, 2000, 1995; Hennink et al., 2017).

### Study Design

A qualitative cross-sectional interview-based study was conducted to identify and describe Deaf ASL users' pharmacy experiences and technology use. A member of the study team pre-screened potential participants via videophone or email to confirm that they met the inclusion and exclusion criteria. The recorded interviews were translated into English, transcribed, and then analyzed using thematic coding analysis. Participation was voluntary, and all participants provided their consent.

The protocol for the interviews consisted of sixteen questions developed for this study. Prior and ongoing work carried out by the research team on medication-related experiences for Deaf individuals informed the experiences to be covered and the wording of questions. The research team also examined questions and responses used in other studies carried out on and with this population covering areas relevant to the present inquiry, as a further guide in structuring and sequencing questions (Ferguson & Liu, 2015; Chong et al., 2019). Questions in the interview protocol were put in final form, through an iterative process and pre-tested with a group of hearing and Deaf experts in health disparities (**Table A)**. All study activities were approved by the University of Rochester Research Subjects Review Board and the Rochester Institute of Technology Institutional Review Board.

**Table A:**
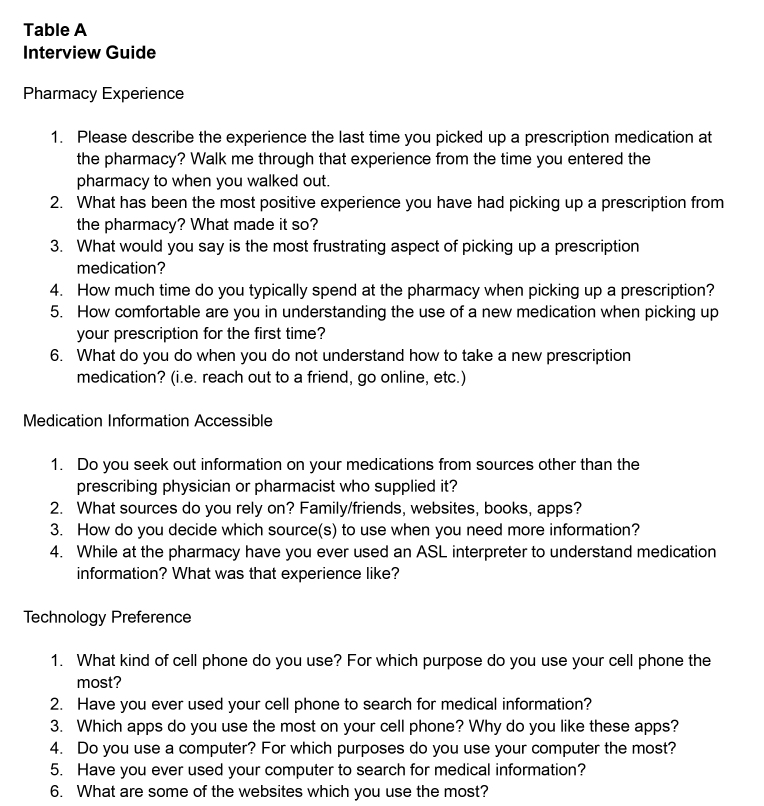
Interview Guide

Pharmacy Experience 1. Please describe the experience the last time you picked up a prescription medication at the pharmacy? Walk me through that experience from the time you entered the pharmacy to when you walked out. 2. What has been the most positive experience you have had picking up a prescription from the pharmacy? What made it so? 3. What would you say is the most frustrating aspect of picking up a prescription medication? 4. How much time do you typically spend at the pharmacy when picking up a prescription? 5. How comfortable are you in understanding the use of a new medication when picking up your prescription for the first time? 6. What do you do when you do not understand how to take a new prescription medication? (i.e. reach out to a friend, go online, etc.)
Medication Information Accessible 1. Do you seek out information on your medications from sources other than the prescribing physician or pharmacist who supplied it? 2. What sources do you rely on? Family/friends, websites, books, apps? 3. How do you decide which source(s) to use when you need more information? 4. While at the pharmacy have you ever used an ASL interpreter to understand medication information? What was that experience like?
Technology Preference 1. What kind of cell phone do you use? For which purpose do you use your cell phone the most? 2. Have you ever used your cell phone to search for medical information? 3. Which apps do you use the most on your cell phone? Why do you like these apps? 4. Do you use a computer? For which purposes do you use your computer the most? 5. Have you ever used your computer to search for medical information? 6. What are some of the websites which you use the most?

### Interviews

A semi-structured 60-minute interview was used to gather information from participants relating to their pharmacy and medication-specific experiences. These interview recordings were conducted in ASL and captured the screens of the interviewer and participant via HIPAA-compliant Zoom. Interview recordings were sent to an ASL-English translation service for transcription. Participants received compensation for their participation.

### Analysis

Based on prior research within the Deaf community, the research team created a list of potential themes that could emerge from the interviews as the pre-set coding scheme. Collectively, the three researchers as a group coded the first English transcript to establish a common understanding of the coding scheme. They independently coded the remaining transcripts. Upon completion, the team reviewed the three sets of codes again as a group to allow for comparisons and discussion of any disagreements. This “negotiated agreement” approach (Campbell et al., 2013) was used with the aim of arriving at a final consensus version of the codes. In addition, when the team was unsure of the meaning of a statement in a transcript, Deaf health experts were consulted for clarification. As other themes emerged from the transcripts, new codes were added. In addition, recurring codes were grouped together to identify themes specifically associated with pharmacy and medication use experiences within the Deaf community.

## Results

### Participants

A total of 25 adults participated in this study. **Table 1** summarizes key characteristics of the study population. Participants self-identified as Deaf (56%), deaf (40%), and hard of hearing (4%). The average age of participants was 44.9 with most participants (32%) being between ages 46 and 55 and the fewest (16%) being age 56 and above. Most participants (68%) identified as women, and one participant identified as non-binary (4%). Education level distribution was: 56% of participants earned 4-year college degrees, 32% earned a Master's degree or above, and 12% completed high school or some college. Participants cited the use of four categories of assistive listening devices: hearing aids (48%), cochlear implants (36%), a combination of hearing aids and cochlear implants (8%), and non-user (8%). Finally, nearly all participants (96%) reported owning a mobile phone.

**Table 1:**
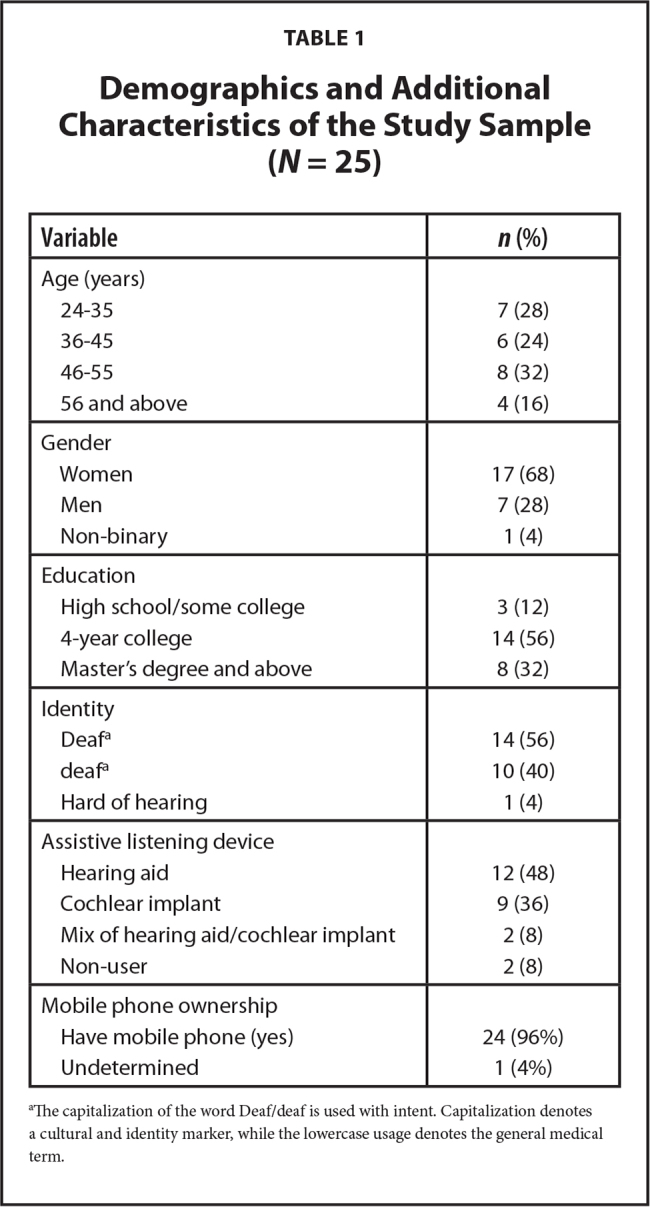
Demographics and Additional Characteristics of the Study Sample (*N* = 25)

**Variable**	***n* (%)**

Age (years)	
24–35	7 (28)
36–45	6 (24)
46–55	8 (32)
56 and above	4 (16)

Gender	
Women	17 (68)
Men	7 (28)
Non-binary	1 (4)

Education	
High school/some college	3 (12)
4-year college	14 (56)
Master's degree and above	8 (32)

Identity	
Deaf^a^	14 (56)
deaf^a^	10 (40)
Hard of hearing	1 (4)

Assistive listening device	
Hearing aid	12 (48)
Cochlear implant	9 (36)
Mix of hearing aid/cochlear implant	2 (8)
Non-user	2 (8)

Mobile phone ownership	
Have mobile phone (yes)	24 (96%)
Undetermined	1 (4%)

a The capitalization of the word Deaf/deaf is used with intent. Capitalization denotes a cultural and identity marker, while the lowercase usage denotes the general medical term.

Throughout the interviews, participants described experiences accessing, making sense of, and utilizing information about prescription medications. The interviews ranged in length from 12 minutes to 43 minutes. This time allowed for exploration of experiences, with comments and responses analyzed for patterns across the interviews. Distinct themes were identified: (1) medication information seeking, (2) comfort taking medication, (3) picking up medication, and (4) communication with the pharmacy team.

### Medication Information Seeking

**Figure 1.**
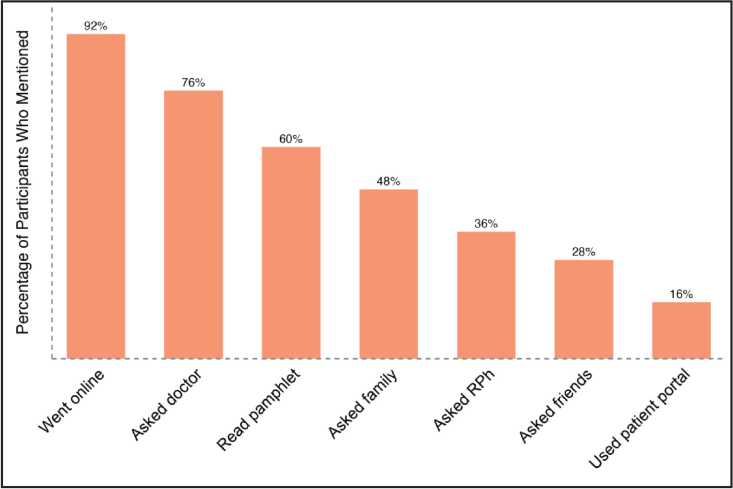
Medication information seeking behavior—Participants (N = 25) were asked the following questions: (1) Do you seek out information on your medications from sources other than the prescriber or pharmacist who supplied it? (2) What sources do you rely on? (3) How do you decide which source(s) to use when you need more information? RPh = pharmacist.

The first theme that emerged from the interviews is where and how individuals seek medication information. **Figure 1** provides the breakdown of the various sources consulted. The comments and responses reveal that those interviewed draw from three types of sources when seeking information about medications; namely online websites, practitioners, and peers (family and friends). The source mentioned most often by participants was websites. The percentage of participants mentioning web searches (92%), including patient portals, exceeds the percentage mentioning questions/requests posed directly to physicians (76%), family (48%), the pharmacy team (36%), or friends (28%). Those interviewed who sought information online referred to websites, namely Google, WebMD, the Mayo Clinic website, and Pills.com.

Beyond consulting websites, participants revealed a reasonable pattern in requests for medication information posed directly to practitioners and others including family and friends. From the time of receiving a prescription to medication pick-up at the pharmacy, the prescriber was most often consulted. At the time of pick-up of the prescribed medication, the pharmacy team was most frequently consulted. During the period from receiving a prescription through pick-up of medication, participants also consulted family or friends. Notably, however, 8% of participants indicated that they do not ask friends or family members about their medications.

### Comfort With Taking Medications

Another theme of the overall experience is comfort in understanding how to use a prescribed medication at the time of pick-up. In conveying levels of comfort in understanding the use of medications when first picked up, those interviewed elaborated on experiences, whether generally favorable or generally unfavorable.

**Figure 2.**
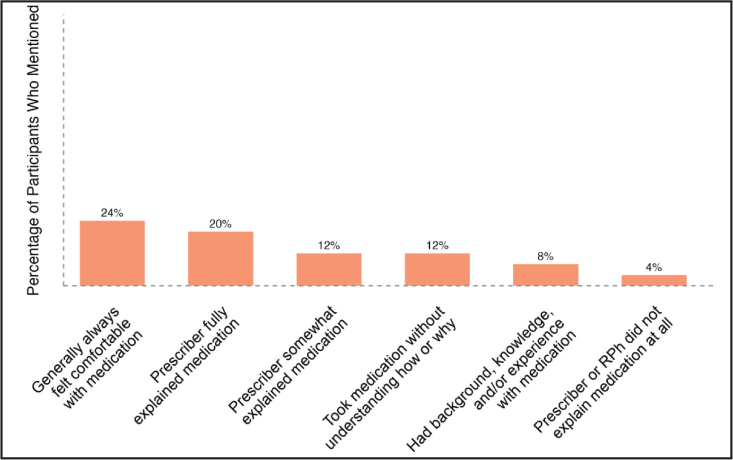
Comfort taking prescription medications—Participants (N = 25) were asked the following questions: (1) How comfortable are you in understanding the use of a new medication when picking up your prescription for the first time? (2) What do you do when you do not understand how to take a new prescription medication? RPh = pharmacist.

These experiences, including some reasoning behind varying levels of comfort associated with medication use, are presented in **Figure 2**. Participants who reported feeling comfortable understanding the use of new medications for the first time commonly mentioned that the prescriber fully explained the new medication at the time of medication being prescribed (20%). The participants less commonly mentioned their own general knowledge or experience with the medication (8%).

Negative experiences related to comfort taking medications were also discussed. Four percent of participants expressed a lack of comfort in understanding the use of prescribed medications when first picked up. Twelve percent reported taking the prescribed medications when they did not understand the purpose of the medication and/or the proper way to use the medication.

### Medication Pick-Up Experiences

**Figure 3.**
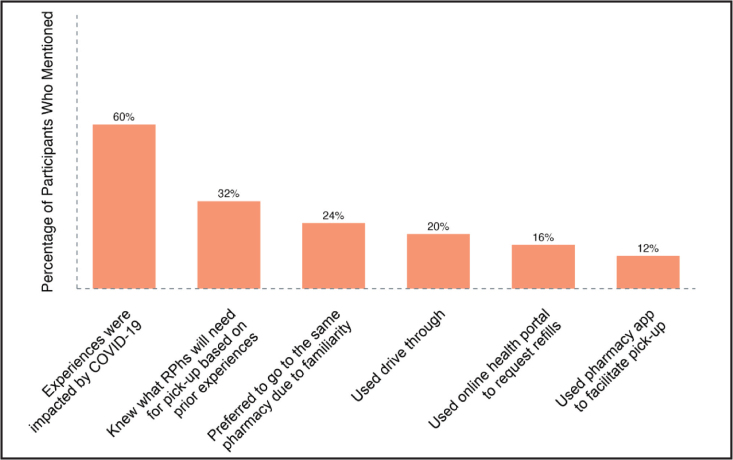
Medications pick-up experiences—Participants (N = 25) were asked the following questions: (1) Please describe the experience the last time you picked up a prescription medication at the pharmacy. Walk me through that experience from the time you entered the pharmacy to when you walked out. (2) What has been the most positive experience you have had picking up a prescription from the pharmacy? What made it so? (3) What would you say is the most frustrating aspect of picking up a prescription medication? RPh = pharmacist.

In a series of questions, we asked participants to describe their experience picking up medications at the pharmacy from start to finish, seen in **Figure 3**. Beginning with the medication refill process, a low number of participants reported using an online resource to monitor and refill their prescriptions. Of those, two options became clear: an online health portal (16%) and a pharmacy-based app, such as that found on the CVS website (12%). Once a medication had been refilled, participants often went to the pharmacy alone (12%) as opposed to sending someone else on their behalf (12%). Upon arrival, 20% of participants noted that they found using the drive through beneficial.

Of those that entered the pharmacy, many reported the importance of their routine of medication pick up. Based on repeated experiences at the same pharmacy, 32% of participants were able to predict what personal information was required for medication pick up and established a routine that they could follow. Familiarity with the specific pharmacy played a large role in this, as participants reported an additional positive experience when the pharmacy team was familiar with them—with some participants attributing this positive experience to the pharmacy team's overall familiarity with DHH individuals. Reported wait times at the pharmacy varied, with the slight majority estimating the wait to be about 15 minutes. 32% of participants reported an overall positive medication pick up experience.

Sixty-eight percent of participants mentioned an impact of coronavirus disease 2019 (COVID-19) on the medication pick-up experience. Most notably, participants described the new barriers to communication that arose in the face of COVID-19, and the resulting changes they made to adapt to the pandemic. More than one-third of participants (36%) mentioned increased difficulty of lipreading when the pharmacy team wore face masks. Further, participants mentioned that interpreting facial expressions (20%) and attempting to hear the pharmacy team (12%) were increasingly difficult during this time. As a result of these barriers, and the reported unwillingness of the pharmacy team to attempt writing back and forth (4%), communication strategies (16%) and medication pick-up behaviors (12%) changed during the pandemic.

### Communication with Pharmacy Team

**Figure 4.**
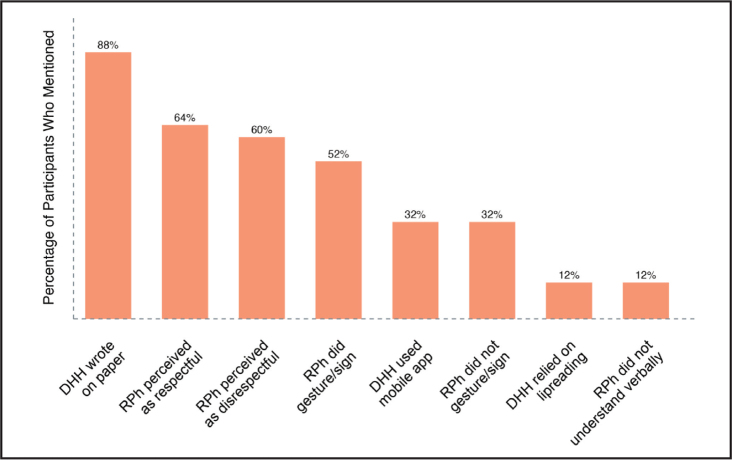
Communication experiences with the pharmacy team—Participants (N = 25) were asked the following questions: (1) Please describe the experience the last time you picked up a prescription medication at the pharmacy? Walk me through that experience from the time you entered the pharmacy to when you walked out. (2) What has been the most positive experience you have had picking up a prescription from the pharmacy? What made it so? (3) What would you say is the most frustrating aspect of picking up a prescription medication? (4) While at the pharmacy have you ever used an American Sign Language interpreter to understand medication information? What was that experience like? DHH = deaf and hard-of-hearing; RPh = pharmacist.

A major point of discussion during the interviews was the DHH experience communicating directly with the pharmacy team. Details of this theme, which include both positive and negative pharmacy team interactions, are presented in **Figure 4**. Eighty-eight percent of participants reported the lack of interpreters at the pharmacy. This forced participants to use the communication strategies outlined in **Figure 4**, which displays the strategies mentioned by the communication method. Among those using their phones to communicate, some appreciated the large and clear text enabled through typing on apps like Transcribe and Onduo (32%). In addition to describing communication strategies, participants commented on the relative success of such methods and their impact on their overall communication experience.

Sixty percent of participants described negative experiences communicating with the pharmacy team during which the team was perceived as disrespectful in the interaction. Further, 12% of participants mentioned the pharmacy team's general inability to understand DHH verbal communication. In addition, while participants expressed a desire to have pharmacists and additional staff proficient in ASL, this was often not the case. In fact, 32% of participants described how the pharmacy team made no attempt to gesture or sign to communicate, further compounding barriers to communication. These negative experiences culminated in some participants electing not to ask the pharmacy team questions about their medications (12%).

Although many participants shared negative communication experiences with the pharmacy team, some did reference positive experiences. These positive experiences centered around the pharmacy team's familiarity with the DHH population.

## Discussion

Given the aforementioned themes identified in the interviews, we can contextualize experiences of participants within real-world barriers of an audiocentric world in which speaking and hearing is the norm (Eckert & Rowley, 2013; Hauser et al., 2010; Wright, 2021). This pilot study is the first known attempt to record how deaf individuals engage with pharmacists for the purpose of medication education and medication adherence; it can be framed in the general sense of barriers that currently manifest in real-world spoken interactions.

Eckert and Rowley (2013) give a simple, but powerful example of a Deaf individual checking out at the grocery store, in which the cashier begins to speak in a typical customer-service script; “How are you, did you find everything you need?” and continues speaking. The deaf customer attempts to gain the cashier's attention by pointing to their ears and shaking their head “no” to indicate that they are deaf. The customer tries to gesture that the cashier can write what they are saying but instead, the cashier replies by shaking their head saying “No, no, that's OK”, abruptly ending the conversation. Eckert and Rowley (2013) argue that the interaction between a Deaf individual and the cashier is defined as aversive audism that deaf people in an array of public interactions unilaterally experienced. Covert institutional audism is defined similarly to aversive racism (Pearson et al., 2009) in which individuals or institutions purport to advance diversity, equity, and inclusion, but the presence of deaf people invariably leads to anxiety and even basic norms of respectful communication are disregarded. The example of a deaf customer at the grocery store can easily be interchanged with a deaf person engaging with a pharmacist, as evidenced by this pilot study.

More importantly, DHH individuals are routinely accustomed to such barriers, as deaf individuals are expected to shift toward an audiocentric center in terms of easing communication for hearing people through lip-reading, or attempting to communicate verbally or nonverbally, but hearing people are not expected to reciprocate with mutual respect (Altieri et al., 2011; Balkany et al., 1996; Davenport, 1977; Loeb, 1993; Sabatello, 2005; Tucker, 2004). Unlike foreign-language barriers, deaf literacy renders medical information functionally obsolete (McKee et al., 2015).

Here, we argue based on the perspectives of participants in this study that strategies of written communication, use of technology, and gesturing fall far short of receiving functionally equivalent access to prescription medication. Taken together, the health literacy of deaf individuals coupled with ingrained aversive attitudes toward deaf individuals and an overall lack of preparedness for pharmacists interacting with deaf individuals presents a battery of challenges for DHH individuals in the access of functionally equivalent medication information as is evident in many study participants (Bailey et al., 2021; Cardosi et al., 2018; Takara et al., 2021).

With the issues of health literacy, and impact of potential aversive audism we can see how the results in **Figure 2** are consistent with the notions of “prescriber or pharmacist does not explain medication at all”, “prescriber somewhat explains medication” or participant “takes the medication without understanding how or why.” A previous study stated that the majority of community pharmacists (83%) have preferred to communicate with DHH patients by writing information (Chong et al., 2021). Crucially, the issue of written information as an accommodation is problematic for two main reasons: (1) written English is generally not the most accessible language for DHH individuals (Cuculick & Kelly, 2003; McKee et. al., 2019); and, (2) the act of writing long-form medication regimens, warnings, and side effects are not functionally equivalent. For the majority of DHH individuals, the most accessible language is ASL whereas complex written English instructions leave more room for misunderstanding and the potential for unintentional misuse of medication potentially leading to fatal results. When hearing individuals interact with their pharmacist, it is sufficient to discover medication instructions, with mutual respect (Bailey et al., 2021; Cardosi et al., 2018). Due to dependence upon aversive audism and provider attitude, a written consultation is a time-consuming barrier. The information disseminated in the process of dispensing a medication is as important as the medication itself, sometimes more important. Therefore, trained pharmacists qualified in intercultural care are key to providing patient-centered pharmaceutical care (Takara et al., 2021).

This work also provides a foundation in understanding the role and impact of technology on medication-related experiences for DHH individuals. Technology was an ubiquitous part of the experience, as evidenced through the interviews. Mobile technology affords portability and convenience, building on an existing comfort and ease of use. While existing mobile applications address some Deaf health related needs, none specifically address medication literacy (Ava, 2020; Chininthom, 2020). Additionally, existing applications do not consistently use visuals, such as pictures and videos in ASL, as a means to present information—a key factor for accessible design. Further work is warranted to explore additional needs and preferences of Deaf ASL users to inform an accessible mobile-based technology solution in support of improved pharmacist communication, and ultimately, medication literacy (Blakely et al., 2020).

The small sample is generalizable to the Deaf population in the northeastern U.S. region. However, results need to be situated in relation to characteristics of the sample. Forty percent of the participants identified as deaf and having an ability to communicate independently. This group is less vulnerable to communication barriers in the pharmacy service. Given that 88% of participants had at least a 4-year college degree, the medication seeking behavior in this sample may not be representative. Due to a technical glitch, two recorded interviews did not include the interviewer's screen. The transcriber could not see the interviewer's questions in ASL, which may have resulted in stymieing the translation accuracy. However, the translation service was provided with all interview questions in written form. The interviewer likely asked the questions in ASL as written. Since the transcriber aligned responses in ASL to the written questions in the transcripts, the two interviews were included in this analysis.

Although this thematic analysis relied on systematic and rigorous techniques by multiple reviewers, there is still subjectivity innately built into qualitative data interpretation. Further, while we did not engage in an internal checking process—sharing the brief findings of our research study with participants to confirm the accuracy of responses (Birt et al., 2016)—we did confirm the context and nature of experiences with those from the Deaf community who were a part of the research team.

## Conclusion

This study has as its contribution a preliminary description of Deaf ASL users' medication-related experiences and technology use within this context. The results and findings fill a gap, as there are no extant studies of such Deaf ASL users' experiences in the U.S. Under themes which broadly capture and describe experiences reported by study participants, our results provide details on the specific and unique manifestations of medication use and interactions with the pharmacy team for the Deaf ASL users. Additionally, we found that technology greatly aided the participants during this experience. Deaf ASL users have unique needs, expectations, and preferences related to accessing and using prescription medications. Future work is needed to continue to explore the potential role of accessible technology in improving medication literacy within the DHH community.
